# Resistance of the target islet tissue to autoimmune destruction contributes to genetic susceptibility in Type 1 diabetes

**DOI:** 10.1186/1745-6150-2-5

**Published:** 2007-01-25

**Authors:** Natasha J Hill, Aleksandr Stotland, Michelle Solomon, Patrick Secrest, Elizabeth Getzoff, Nora Sarvetnick

**Affiliations:** 1 Department of Immunology, The Scripps Research Institute, La Jolla, California, USA; 2 Department of Molecular Biology and the Skaggs Institute for Chemical Biology, The Scripps Research Institute, La Jolla, California, USA; 3 Centre for Diabetes and Metabolic Medicine, Institute of Cell and Molecular Sciences, Barts and the London Queen Mary's School of Medicine and Dentistry, London, UK

## Abstract

Type 1 diabetes occurs when self-reactive T lymphocytes destroy the insulin-producing islet β cells of the pancreas. The defects causing this disease have often been assumed to occur exclusively in the immune system. We present evidence that genetic variation at the *Idd9 *diabetes susceptibility locus determines the resilience of the targets of autoimmunity, the islets, to destruction. Susceptible islets exhibit hyper-responsiveness to inflammatory cytokines resulting in enhanced cell death and increased expression of the death receptor Fas. Fas upregulation in β cells is mediated by TNFR2, and colocalization of TNFR2 with the adaptor TRAF2 in NOD β cells is altered. *TNFR2 *lies within the candidate *Idd9 *interval and the diabetes-associated variant contains a mutation adjacent to the TRAF2 binding site. A component of diabetes susceptibility may therefore be determined by the target of the autoimmune response, and protective TNFR2 signaling in islets inhibit early cytokine-induced damage required for the development of destructive autoimmunity.

This article was reviewed by Matthiasvon Herrath, HaraldVon Boehmer, and Ciriaco Piccirillo (nominated by Ethan Shevach).

## Open peer review

Reviewed by Matthiasvon Herrath, HaraldVon Boehmer, and Ciriaco Piccirillo (nominated by Ethan Shevach). For the full reviews, please go to the Reviewers' comments section.

## Background

The autoimmune nature of type 1 diabetes has led to a focus on cells of the immune system in the search for defects that underlie genetic predisposition [1]. Overt diabetes is preceded by an inflammatory islet infiltration, known as insulitis, that results in the targeted deletion of insulin-producing β cells and subsequent loss of glucose homeostasis. However, insulitis does not inextricably lead to islet destruction, even if significant damage is inflicted. Cytokines such as TNF, IFNγ and IL1β released by inflammatory cells within islets play an important role in sensitizing β cells to apoptosis and cell death [2]. However, in non-autoimmune prone individuals, initial islet infiltration and cytokine release may promote repair and regeneration. An aberrant response to inflammatory cytokines on the part of islets during a critical early stage of autoimmunity may therefore contribute to diabetes susceptibility.

Both CD4 and CD8 T cells present in the inflammatory lesion have the potential to cause β cell loss and both are required for spontaneous diabetes [3]. MHC Class I-restricted recognition of β cells by CD8 T cells is crucial for the transition from insulitis to diabetes [4]. Perforin and Fas-mediated cell death both play a role in CD8-mediated islet destruction [5]. The targeted release of TNF and IFNγ upon class I recognition induces islet expression of Fas [6,7], as well as MHC class I, immunoproteosome subunits [8], and also cytokines such as IL15 [9] and chemokines such as CXCL10/CXCL9 [10] that promote T cell survival and recruitment. The release of inflammatory cytokines within islets can therefore promote islet inflammation and cell death, but islets may perhaps normally be able to regulate pathogenic changes [11].

There is currently limited understanding of how the progression of insulitis to diabetes can be regulated. It has been shown previously that non-obese diabetic (NOD) mice expressing B10 resistance alleles at the *Idd9 *genetic susceptibility locus, NOD.B10*Idd9 *congenic mice (*Idd9 *congenic mice), are highly protected against diabetes [12]. *Idd9 *genes appear to control the progression from infiltration to islet destruction, and it was proposed that protective *Idd9 *genes cause the priming of a non-pathogenic autoimmune response [12]. Establishing the mechanisms by which protective physiological variants of diabetes susceptibility genes, such as *Idd9*, prevent diabetes is important for understanding how genetic variation influences disease susceptibility, and also to discover the natural points of control at which disease progression can be averted.

We have discovered evidence that diabetes protection mediated by *Idd9 *genes is localized to the target islet tissue itself. The islet infiltrate in *Idd9 *congenic mice contains fewer CD8 T cells, and islets from *Idd9 *congenic mice are resistant to CD8 T cell mediated destruction. *Idd9 *congenic islets demonstrate altered TNF/IFNγ responsiveness *in vitro*, with less cell death and reduced Fas expression compared to NOD islets following cytokine exposure. We show that TNFR2, and also the TNFR2 signalling adaptor proteins, TRAF2 and RIP, are expressed in islets, and that TNFR2-deficient islets are defective in their ability to upregulate Fas following TNF/IFNγ exposure. TNFR2 signalling is therefore important in mediating TNF-responses in islets. Furthermore, blocking TNFR2 in *Idd9*, but not NOD, islets inhibits cytokine-induced Fas upregulation, and the TNF-induced colocalization of TRAF2 with TNFR2 is prolonged in NOD islet β cells. These results together suggest that the termination of TNFR2 signalling in NOD islets may be defective, and that this trait is corrected by *Idd9 *resistance genes. This raises the possibility that protective islet TNFR2/TRAF2 signaling may confer resistance to islet destruction and diabetes. An important implication of these results is that islet defects can contribute to Type 1 diabetes susceptibility, and that promoting protective islet responses to cytokines may be effective in preventing recurrent autoimmune disease and improve the success of islet tissue replacement therapies.

## Results

### The islet infiltrate in *Idd9 *congenic mice contains a reduced % CD8 T cells

**Figure 1:**
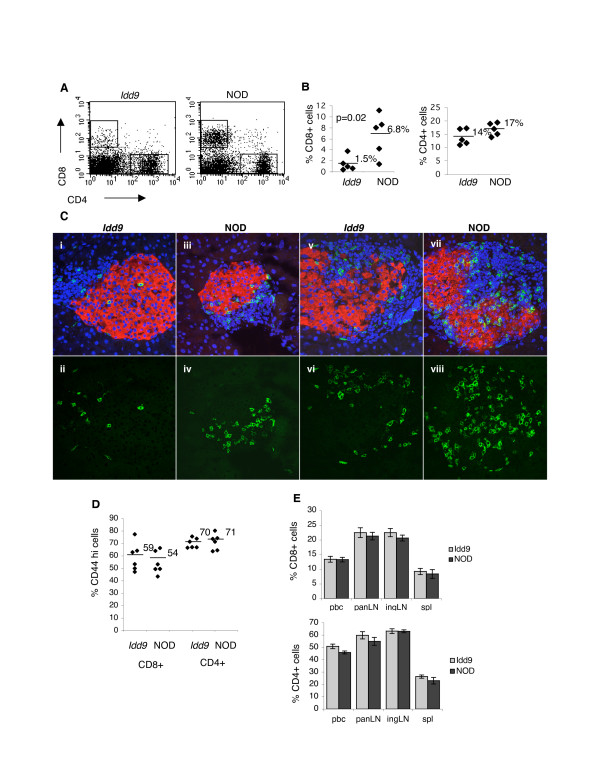
**Reduced % CD8 T cells in the *Idd9 *congenic islet infiltrate**. Islet infiltrating cells were isolated from the islets of *Idd9 *congenic and NOD mice for analysis by flow cytometry. Dot plots showing representative CD4 and CD8 staining, gated on the CD45+ population (A), and the range of values for %CD8+ and %CD4+ cells (B) are also shown. Results representative of > 6 independent experiments (mice aged 12–14 weeks). The presence of CD8+ cells in the islets of *Idd9 *congenic and NOD mice was also determined by confocal microscopy of frozen pancreatic sections (C). Sections (10 μm) from 12–14 week old mice were stained with antibodies to CD8 (green) and insulin (red), and Topro-3 (blue) was used to visualize nuclei. A total of 27 infiltrated islets from 4 *Idd9 *congenic mice, and 36 infiltrated islets from 4 NOD mice were examined. The islets were imaged from three different levels through each pancreas, each level separated by at least 300 μm. Lower panels show fluoroscein staining only to aid comparison of CD8 infiltration between *Idd9 *congenic and NOD islets. Islets from the two strains exhibiting similar areas of infiltration were compared (i-iv show minor infiltration, and v-viii an extensive infiltrate). Original magnification 40 ×. In (D), flow cytometry staining of the activation marker CD44 on CD4 and CD8 T cells isolated from the islet infiltrate of 12–14 week old mice is shown. Bars and adjacent numbers in plot indicate average value. n = 6, representative of 2 independent experiments. The % CD4 and CD8 T cells in peripheral blood and secondary lymphoid organs of 6 week old *Idd9 *congenic mice was also determined by flow cytometry (E). Average values +/- SEM are shown. n = 4, representative of 2 independent experiments.

*Idd9 *congenic mice develop a high degree of insulitis yet very few go on to develop diabetes [12]. To test for differences in the cellular composition of *Idd9 *islet infiltrates we quantified the major leukocyte populations present in the infiltrate of age-matched 12–14 week old *Idd9 *congenic and NOD mice by flow cytometry. We observed a striking reduction in the % CD8 T cells in the *Idd9 *congenic infiltrates (p = 0.02), as shown in Table 1 and Figure 1(A–B). The reduction in CD8 T cell infiltration was also evident *in situ*, by staining pancreatic sections (Figure 1C). Expression of the activation/memory marker CD44 was equivalent in both CD4 and CD8 T cell populations within the islet infiltrate (Figure 1D) suggesting that the activation state of T cells recruited to the islets is unaffected in *Idd9 *congenic mice. No significant difference in the % CD8 T cells in secondary lymphoid organs or peripheral blood was observed in *Idd9 *congenic mice either at 6 weeks of age (Figure 1E), or in older mice (data not shown). Therefore, the decreased % CD8 T cells is specific to the islet environment.

**Table 1:** Characterization of major cell types in the *Idd9 *islet infiltrate

		**Mean**		**Sem**	
Gate		*Idd9*	NOD	*Idd9*	NOD
	%CD45	54.2	51.8	4.4	10.0

CD45+	%CD11b	30.8	26.3	3.2	6.2
CD45+	%CD11c	9.9	9.9	1.2	2.9
CD45+	%B220	42.7	36.5	4.0	6.5
CD45+	%CD4	14.0	16.9	1.3	1.0
CD45+	**%CD8**	**1.5**	**6.8**	0.6	1.7

Islets isolated from the pancreas of 12 week old female *Idd9 *congenic (n = 5) and NOD (n = 5) mice using histopaque density gradient and stained for analysis by flow cytometry to identify the major infiltrating leukocyte populations. Staining for CD45 was used to gate on the leukocyte population. Representative of two independent experiments.

### Islet-reactive CD8 T cells are able to become activated in the panLN of *Idd9 *congenic mice

**Figure 2:**
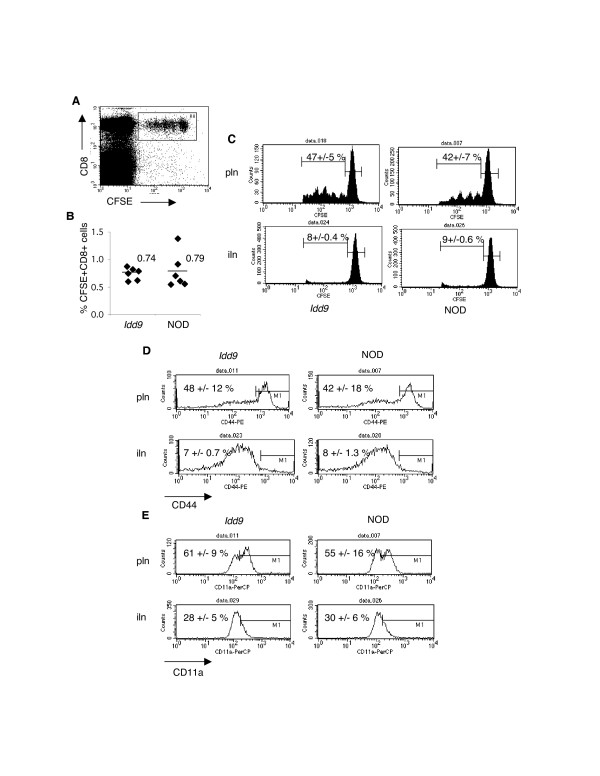
**Equivalent expansion and activation of islet specific CD8 T cells in the panLN of *Idd9 *congenic and NOD mice**. CFSE-labeled splenocytes (20 million cells) from 4–6 week old 8.3NOD*Scid *donor mice were transferred into age-matched 6–9 week old *Idd9 *congenic and NOD recipients. On day 4 following transfer panLN and ingLN cells were stained for analysis by flow cytometry. Donor islet-specific CD8 T cells were defined by gating on CFSE+CD8+ cells (A). The percentage of CFSE+CD8+ T cells recovered from the panLN of *Idd9 *congenic and NOD mice is shown in (B). Representative CFSE profiles of CFSE+CD8+ cells in the panLN and ingLN, with the average % divided cells +/- SEM, are shown in (C). We also stained for the activation markers CD44 (D) and CD11a (E), and representative histograms (gated on CFSE+CD8+ cells) are shown for *Idd9 *congenic and NOD panLN and ingLN cells, indicating the average value +/- SEM. Data is representative of 4 independent experiments n = 5–6 mice for each experiment.

Having found that CD8 T cell infiltration is reduced in *Idd9 *congenic islets we wanted to test whether the activation of islet-specific CD8 T cells in lymph nodes is inhibited in *Idd9 *congenic mice. We transferred CFSE-labeled splenocytes from a TCR transgenic strain (8.3NOD*Scid*) [13] expressing islet-specific CD8 T cells into *Idd9 *congenic and NOD recipients. The CFSE+CD8+ (Figure 2A) populations recovered from the panLN of *Idd9 *congenic and NOD recipients were numerically equivalent (Figure 2B) and underwent similar cell division, primarily in the panLN where the cognate antigen is present (Figure 2C). We also examined the expression of activation markers on adoptively transferred 8.3 T cells. CFSE+CD8+ cells expressed high levels of both CD44 (Figure 2D) and CD11a (Figure 2E) specifically in the panLN. However, there was no difference in the expression of CD44 and CD11a on transferred 8.3 T cells in *Idd9 *congenic and NOD mice. The activation of islet-specific CD8 T cells on the NOD background is therefore not inhibited in the *Idd9 *panLN.

### *Idd9 *congenic mice retain diabetes protection in the presence of NOD-derived immune cells, yet there is no evidence of immune regulation

**Figure 3:**
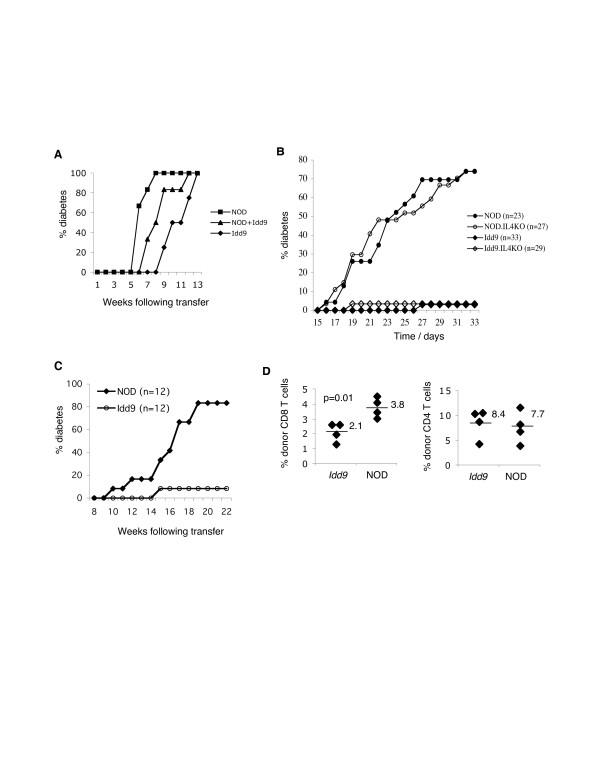
***Idd9 *congenic mice retain diabetes protection in the presence of NOD-derived bone marrow, yet no evidence of immune regulation**. (A) Diabetes incidence in NOD*Scid *recipients following adoptive transfer of 20 million splenocytes from 13 week old NOD (n = 6) and *Idd9 *congenic (n = 4) mice, or co-transfer of 10 million NOD plus 10 million *Idd9 *congenic splenocytes (n = 6) determined by weekly blood glucose monitoring. The results are representative of 2 experiments. (B) Blood glucose levels measured weekly from 15 weeks of age in female NOD (n = 23), NOD.IL4KO (n = 27), *Idd9 *congenic (n = 33), and *Idd9*.IL4KO (n = 29) mice until 33 weeks of age to determine diabetes incidence. In (C), diabetes incidence is shown for lethally irradiated (950 Rad) *Idd9 *congenic and NOD mice reconstituted with bone marrow cells (10 million) from 5 week old NOD donors. Results representative of 2 independent experiments. In (D) bone marrow cells (10 million) from NOD.NONThy1.1 donors were injected as in (C). Islet-infiltrating cells were isolated from recipients 6 weeks following transfer and the % donor (Thy1.1+) CD4 and CD8 T cells determined by flow cytometry.

To test whether the diabetogenic potential of the anti-islet immune response is reduced in *Idd9 *congenic mice we transferred splenocytes from 13 week old *Idd9 *congenic and NOD donors into NOD*Scid *recipients, or co-transferred 10 million cells from each strain, and monitored the incidence of diabetes. The transfer of both *Idd9 *congenic and NOD cells individually induced diabetes in 100 % of recipients (Figure 3A), demonstrating that *Idd9 *splenocytes have the capacity to induce disease. However, the onset of disease was delayed in the recipients of *Idd9 *cells (p = 0.002). *Idd9 *splenocytes are therefore fully capable of inducing diabetes, but do so with delayed kinetics compared to NOD splenocytes. The kinetics of diabetes onset in co-transferred recipients was intermediate between that seen in recipients of either type of splenocytes alone, demonstrating that although *Idd9 *congenic splenocytes are less pathogenic, there is no evidence that they are able to regulate diabetes induction. This conclusion is further supported by the fact that the diabetes incidence induced by 10 million NOD splenocytes was less at 13 weeks post-transfer (67 %, n = 6, data not shown) than the 100 % diabetes induced by co-transfer of 10 million NOD plus 10 million *Idd9 *splenocytes.

It had previously been suggested that the reason *Idd9 *congenic mice are protected against diabetes, despite having an extensive islet infiltrate, could be due to the priming of a non-pathogenic anti-islet T cell response that releases the protective cytokine IL4 [12]. To test whether IL4 is required for the protection mediated by *Idd9 *genes we bred *Idd9 *congenic mice deficient in IL4 (*Idd9*.IL4KO mice). As shown in Figure 3B, a high incidence of diabetes was seen in both NOD (74 % diabetes, n = 23) and NOD.IL4KO mice (74 %, n = 27), as previously described [14]. However, *Idd9*.IL4KO mice developed the same low frequency of diabetes (3 %, n = 29) as *Idd9 *congenic mice (3 %, n = 33), demonstrating that IL4 expression is not required for diabetes protection in the *Idd9 *congenic strain.

We then wanted to test whether *Idd9 *protection maps to radiation-sensitive cells of the immune system, or to non-immune cells. We lethally irradiated (950 Rad) young *Idd9 *congenic and NOD mice and reconstituted them with bone marrow from 6 week old NOD mice. Approximately 70 % chimerism was achieved using this protocol [see Additional file 1], therefore while some host hematopoetic cells remain the majority are donor derived. The degree of chimerism was equivalent in the two recipient strains, therefore previous reports describing the competitive advantage of NOD hematopoetic stem cells in allogeneic recipients are not relevant in this case [15]. Whereas 83 % (n = 12) of NOD mice developed diabetes by 20 weeks following bone marrow transfer, only 8 % (n = 12) of *Idd9 *congenic mice developed diabetes (p = 0.0004, Figure 3C). *Idd9 *congenic mice therefore retain their protection against diabetes in the presence of NOD-derived immune cells. Since no evidence of immune regulation was observed in Figure 3A, this suggests that *Idd9 *resistance may map to non-lymphoid cells.

To determine whether the chimeric mice exhibit the same characteristics of disease protection as *Idd9 *congenic mice, we used NOD mice expressing the Thy1.1 allele (NOD.NONThy1.1 mice) as bone marrow donors and tracked the infiltration of donor CD8 T cells in islets. Six weeks following irradiation and bone marrow reconstitution, we isolated islet-infiltrating cells from recipients. While there was no difference in the % Thy1.1+CD4+ cells in the islets of *Idd9 *congenic and NOD recipients, the %Thy1.1+CD8+ cells was significantly reduced (p = 0.01) in the islets of *Idd9 *congenic compared to NOD mice (Figure 3D). Both the reduced % CD8 T cells in the islets of *Idd9 *congenic mice and the protection against diabetes therefore appear to correlate with expression of *Idd9 *gene products in radiation-resistant cells.

### Transplanted *Idd9 *congenic islets are resistant to destruction by islet-specific CD8 T cells

**Figure 4:**
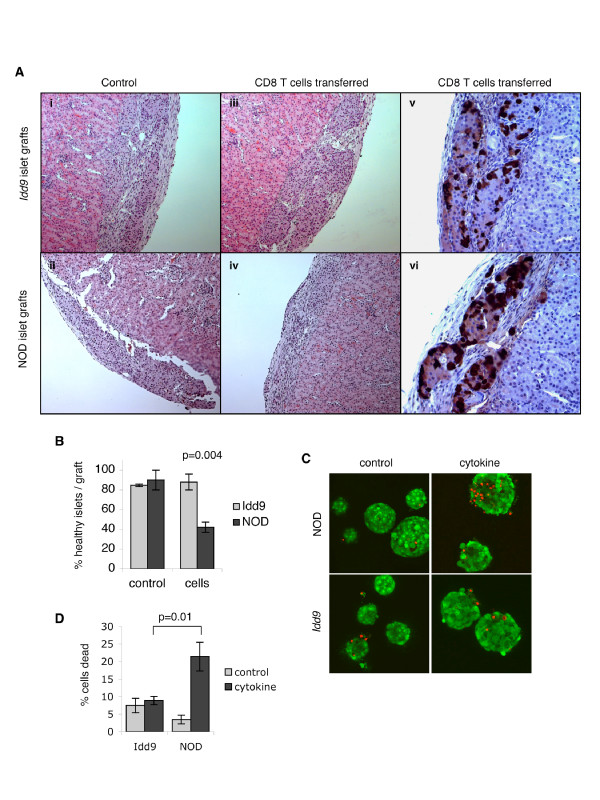
**Islets from *Idd9 *congenic mice are intrinsically resistant to cytokine and CD8 T cell-mediated autoimmune destruction**. Islets isolated from 5 week old *Idd9 *congenic and NOD donors were transplanted under the kidney capsule of NOD*Scid *recipients. After allowing 7 days for the grafts to revascularize, splenocytes from 8.3NOD*Scid *mice (30 million cells) were adoptively transferred into graft recipients. Control mice did not receive splenocytes. Five days later, graft destruction was analyzed by H&E staining of graft sections, as shown in (A) (i-iv). Scoring of graft destruction (B) was determined by anti-glucagon staining (A) (v-vi) as described in the methods. NOD grafts (n = 4), NOD control grafts (n = 2), *Idd9 *congenic grafts (n = 3) and *Idd9 *control grafts (n = 2) were assessed, and a total of 24, 11, 26 and 32 islets scored, respectively, for each group. Graph shows average % healthy islets in each graft +/- SEM. Results representative of 3 independent experiments. (C) Confocal live/dead viability staining of islets from 5 week old *Idd9 *congenic and NOD mice treated with, or without, TNF (2,000 U/ml) and IFNγ (1,000 U/ml) for 6 days. Live cells stain green, and the nuclei of dead cells stain red. For each assay, 7–9 islets (250–500 cells) were analyzed and the average % dead cells per field +/- SEM is shown in (D). Results representative of 3 independent experiments.

The results of the bone marrow reconstitution experiments suggested the possibility that at least a component of the protective effect of *Idd9 *genes may be mediated by non-immune cells. To determine whether protection is localized to the islet tissue we tested whether islets from *Idd9 *congenic mice are resistant to autoimmune destruction. Since we observed a reduced % CD8 T cells in *Idd9 *congenic islets, we hypothesized that islet resistance to CD8-mediated autoimmunity may be specifically affected. We transplanted islets isolated from 5 week old *Idd9 *congenic and NOD mice under the kidney capsule of immunodeficient NOD*Scid *recipients. Islets from young mice with minimal insulitis damage were used as donors and the islets were cultured for several days before transplant to deplete tissue-resident leukocytes. The transplanted mice were injected with splenocytes from 8.3NOD*Scid *TCR transgenic mice and then five days later grafts were taken and the extent of destruction determined by histology (Figure 4A). We observed that *Idd9 *islet tissue remained intact while the 8.3 CD8 T cells destroyed NOD islets. To quantitate the extent of graft destruction we scored the number of healthy islets in each graft using glucagon staining to reveal the presence of islet remnants. Healthy islets show the typical distribution of glucagon-positive cells scattered around the islet periphery, whereas islets in which the core of target insulin-producing cells has been destroyed exhibit a collapsed glucagon-positive remnant. Control grafts, in recipients where no CD8 T cells were transferred, from both *Idd9 *congenic and NOD donors were healthy. In grafts from mice that were injected with islet-specific CD8 T cells, on average only 42 % of NOD islets, compared to 88 % *Idd9 *congenic islets (p = 0.004), were scored as healthy (Figure 4B). Islets from *Idd9 *congenic mice therefore possess an intrinsic capacity to resist CD8-mediated autoimmune destruction.

### *Idd9 *congenic islets exhibit reduced sensitivity to the inflammatory cytokines TNF and IFNγ

NOD islets can be induced to undergo cell death *in vitro *by treatment with TNF in combination with IFNγ [16]. In order to determine whether cytokine-responsiveness is altered in *Idd9 *congenic islets we tested whether sensitivity to *in vitro *cytokine-induced cell death is reduced. Since islet cells can undergo cell death when dispersed into single cells we chose to stain live intact islets with a fluorescent live/dead viability stain and electronically quantitate the % dead cells from confocal images. As shown in Figure 4C–D, cytokine treatment induced a significantly greater amount of cell death in NOD islets compared to *Idd9 *islets (p = 0.01). Islets from *Idd9 *congenic mice therefore display altered cytokine-responsiveness compared to NOD and are resistant to the cytotoxic effects of TNF/IFNγ treatment *in vitro*.

### TNFR2 mediates β cell upregulation of the death receptor Fas in response to cytokines

**Figure 5:**
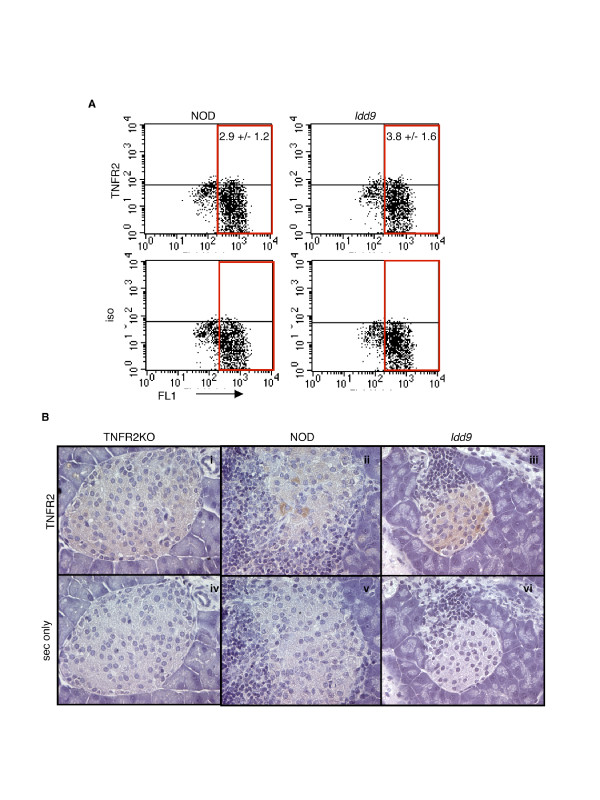
**Detection of TNFR2 expression in islet cells**. (A) Flow cytometry staining with anti-TNFR2 antibody and isotype control in islets isolated from 5 week old mice and cultured for 4–5 days. Auto-fluorescence in the FL1 channel was used to gate on the β cell population, and dead cells were excluded using 7AAD. The gated cells were negative for the hematopoetic cell marker CD45. Values given are the average %TNFR2+ cells +/- SEM (n = 4 each strain), gated on FL1+ cells and corrected for background isotype control staining. Results representative of 3 independent experiments. (B) Immunohistochemical staining of paraffin sections from TNFR2KO (i, iv), 12–14 week old NOD (ii, v) and *Idd9 *congenic (iii, vi) pancreas stained with antibodies to TNFR2 (i-iii) or with the secondary antibody alone (iv-vi). Pancreatic sections from 3 different *Idd9 *congenic and NOD mice, and 1 B6.TNFR2KO mouse, were examined.

The *TNFR2 *gene lies within the *Idd9 *congenic interval and the NOD allele generates a protein with 5 amino acid variants compared to the diabetes resistant B6 strain [17]. *TNFR2 *is therefore a potential candidate gene for the altered responsiveness to TNF/IFNγ we observe in *Idd9 *islets. *TNFR2 *mRNA expression has been shown to be induced in islet cells during diabetes progression [18]. To test whether TNFR2 protein is expressed in islet β cells of diabetes susceptible and resistant strains, we analysed dispersed islets from 5 week old NOD and *Idd9 *congenic mice by flow cytometry. Using the characteristic autofluoresence in the FL1 channel as a β cell marker [19], we found that approximately 3 % β cells are TNFR2 positive (Figure 5A). Immunohistochemical staining of pancreatic sections from 12 week old *Idd9 *congenic and NOD mice confirmed that a sub-population of islet β cells in both strains express TNFR2 protein, whereas no staining occurred using the secondary antibody alone, or in islets from B6.TNFR2KO mice (Figure 5B).

**Figure 6:**
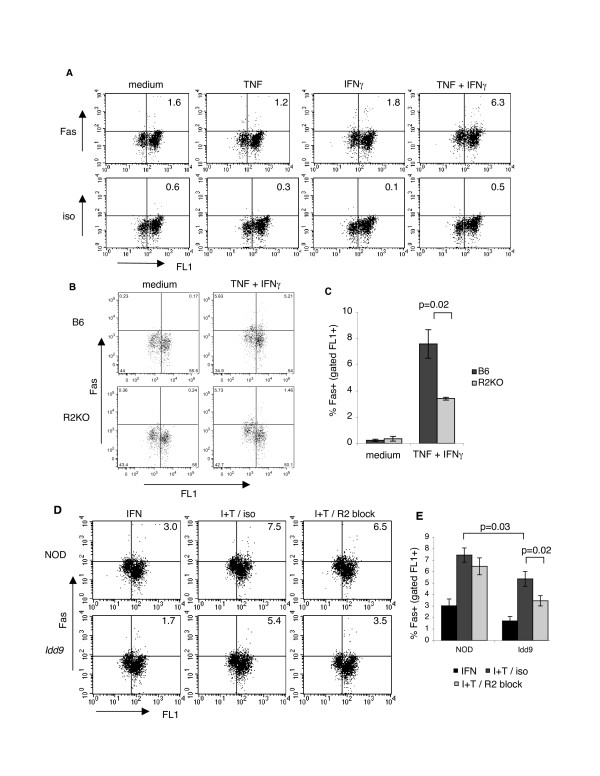
**TNFR2 mediates Fas upregulation, and blocking TNFR2 inhibits Fas upregulation in *Idd9 *congenic but not NOD islets**. (A) Islet Fas expression is induced only by combined TNF+IFNγ treatment. Intact islets from B6 mice were treated with medium, 1,000 U/ml IFNγ and 1,000 U/ml TNF, alone and in combination, for 48 hours. Islets were then dispersed into single cells and, after 60 min recovery incubation, stained with antibodies to Fas or the relevant isotype control for anlysis by flow cytometry. The average %Fas+ cells for each treatment, gated on live FL1+ β cells, is shown. (B) TNFR2 mediates Fas upregulation in β cells. Islets from B6 and B6.TNFR2KO mice (pooled from 2 mice for each strain) were treated in triplicate with medium or IFNγ +TNF (both at 1,000 U/ml) for 48 hours and stained with antibodies to Fas as in (A). Control samples were stained with the relevant isotype control. The % Fas+ cells +/- SEM, gated on live FL1+ β cells, is shown in (C). Representative of 4 independent experiments. (D) Blocking TNFR2 inhibits the upregulation of Fas in β cells from *Idd9 *congenic but not NOD mice. Islets from 5 week old *Idd9 *congenic and NOD mice were treated as before, except that IFNγ +TNF treated islets were pre-incubated for 60 min with 2 μg/ml blocking anti-TNFR2 antibody (I+T+R2), or the relevant isotype control (I+T). Representative dot plots are shown and values indicate the average %Fas+ cells (isotype corrected), gated on live FL1+ β cells, and this data +/- SEM is plotted in (E). The data was pooled from 6 separate experiments with total n = 13–14.

One of the key changes that occurs in β cells in response to cytokines is the upregulation of the death receptor Fas [20], and Fas upregulation may play a role in the process of autoimmune islet cell death *in vivo*. We therefore tested whether TNFR2 expression in islets is required for Fas upregulation following cytokine treatment. Intact islets from B6 mice were treated for 48 hours with medium, TNF, IFNγ, or with a mixture of TNF and IFNγ, then dispersed and stained with antibodies to Fas or isotype control for analysis by flow cytometry. Fas induction occurred only in the presence of IFNγ and TNF, not with either cytokine alone (Figure 6A). To test the role of TNFR2 in Fas induction, we then treated islets from B6 and B6.TNFR2KO with TNF/IFNγ, or with medium alone. As shown in Figure 6B–C, Fas upregulation following IFNγ/TNF treatment was signficantly reduced in B6.TNFR2KO islets compared to B6 islets (p = 0.0003). Fas upregulation was not completely abolished in B6.TNFR2KO islets following TNF/IFNγ-treatment, therefore it is likely that TNFR1 also contributes to Fas upregulation. However, TNFR2 clearly mediates Fas upregulation in β cells in response to cytokines.

### Blocking TNFR2 inhibits Fas upregulation in *Idd9*, but not NOD islets

We therefore tested whether Fas induction is also reduced in *Idd9 *congenic islets. Intact islets from 5 week old donors were again used to minimize insulitis damage and were treated with IFNγ, or a mixture of IFNγ and TNF. As shown in Figure 6D–E, Fas expression was significantly greater in NOD compared to *Idd9 *islets (p = 0.03) following treatment with IFNγ/TNF, further supporting the idea that NOD islets exhibit an enhanced responsiveness to cytokines and that this trait is corrected by expression of protective *Idd9 *gene variants. To test whether TNFR2 signals are involved in mediating this this enhanced responsiveness in NOD islets, we also pretreated islets with a blocking anti-TNFR2 antibody, or with an isotype control, to test whether this would ablate the increased cytokine responsiveness in NOD islets. While Fas upregulation was significantly inhibited in *Idd9 *islets by addition of the blocking TNFR2 antibody (36 % decrease, p = 0.02), Fas induction was only slightly decreased in NOD islets by the presence of the blocking antibody (13 % decrease). This suggests that TNFR2-mediated Fas upregulation in NOD islets is refractive to inhibition compared to *Idd9 *islets.

### Colocalization of TRAF2 with TNFR2 is impaired in NOD islet β cells

**Figure 7:**
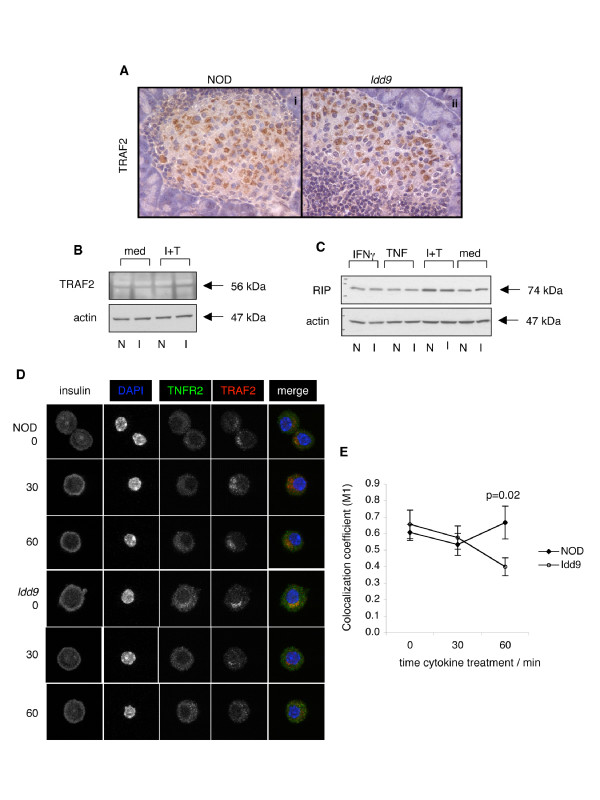
**Co-localization of TRAF2 and TNFR2 following cytokine treatment is prolonged in NOD β cells**. Expression of the adaptor molecules TRAF2 and RIP in islets. (A) Immunohistochemical staining of pancreas sections from 12–14 week old NOD and *Idd9 *congenic mice with anti-TRAF2 antibody. Immunoblotting with antibodies to TRAF2 (B) and RIP (C) of protein lysates prepared from 5 week old *Idd9 *congenic (I) and NOD (N) islets stimulated for 3 days +/- 1,000 U/ml TNF + 1,000 U/ml IFNγ. In each case, membranes were stripped and re-blotted with antibodies to actin as loading control. Results representative of 2–3 independent experiments. (D) Co-localization of TRAF2 and TNFR2 following cytokine treatment determined by confocal imaging. Representative images of β cells from 5 week old NOD and *Idd9 *congenic mice treated with 1,000 U/ml IFNg + 1,000 U/ml TNF for 0, 30 and 60 minutes, stained with antibodies to TNFR2, TRAF2 and insulin, and the nuclear dye DAPI. The merged image of TNFR2 (green), TRAF2 (red) and DAPI (blue) is also shown. Dead cells were excluded using an EtBr-derived dye. In (E) the average TNFR2/TRAF2 colocalization coefficient (M1) +/- SEM is shown for each time point. TNFR2 positive cells are rare within the islet population, but on average 18 TNFR2 positive cells were analyzed per treatment. The threshold for TNFR2 and TRAF2 staining was determined by staining an aliquot of the cells in parallel minus each primary antibody.

TNFR2 signaling in other cell types is known to be mediated via adaptor proteins, particularly RIP and TRAF2, that link the cytoplasmic domain of the receptor to downstream intracellular signaling molecules [21]. We therefore determined whether these adaptors are expressed in islets from *Idd9 *congenic and NOD mice, and tested whether cytokine treatment differentially affects their expression. Immunohistochemical staining of pancreatic sections from 12 week old mice with antibodies to TRAF2 revealed a distinctive staining pattern concentrated in a peri-nuclear compartment (Figure 7A). We then showed by immunoblotting that both TRAF2 (Figure 7B) and RIP (Figure 7C) are expressed in isolated islets. Treatment of intact islets with TNF/IFNγ for 2 days increased the amount of RIP present by approximately 2-fold, while TRAF2 expression was not altered. However, significant differences in the expression of adaptor molecules between *Idd9 *congenic and NOD islet lysates were not observed. Therefore, while differential signaling downstream of TNFR2 may occur in NOD and *Idd9 *congenic islets, it is not likely to be primarily exerted at the absolute level of RIP or TRAF2 protein.

The NOD variant of TNFR2 contains a mutation (C436Y) [17] adjacent to the TRAF2 consensus binding motif (426-SXEE-429) [22] and flanking sequences are thought to modulate TRAF2 binding [23]. We therefore tested whether altered recruitment of TRAF2 by TNFR2 variants could potentially explain the differential responsiveness to TNF/IFNγ that we observed in the islets of *Idd9 *congenic mice. We examined the kinetics of colocalization of TNFR2 and TRAF2 in dispersed islet cells following cytokine stimulation *in vitro*. Dispersed islet cells isolated from young donors were treated with TNF and IFNγ for 0, 30 and 60 minutes and stained sequentially with antibodies to TNFR2, TRAF2 and insulin for analysis by confocal microscopy. DAPI was used as a nuclear dye, and an ethidium bromide derivative used to exclude dead cells from the analysis. Additional cells were stained in parallel minus either the TNFR2 or TRAF2 primary antibody and non-specific staining was not observed. These controls were also used to set the staining threshold for analysis. The colocalization coefficient (M1) of TNFR2 with TRAF2 was then determined for insulin positive TNFR2 expressing β cells at each timepoint. The majority of cells were insulin positive and very few dead cells were observed. TNFR2 positive cells were defined as more than 10 pixels above threshold per cell.

At the zero and 30 minute timepoints the colocalization coefficient in both *Idd9 *congenic and NOD cells was equivalent (approximately 0.6), as shown in Figure 7D–E. At these timepoints, TRAF2 was seen to be primarily concentrated in a peri-nuclear compartment in both strains. TRAF2 remained confined to the peri-nuclear compartment in NOD islet cells at 60 minutes. However, after 60 minutes of cytokine treatment few *Idd9 *cells exhibited this staining pattern and a weak diffuse cytoplasmic staining was more commonly observed instead. Our quantitative analysis indicated that at the 60 minute timepoint the colocalization coefficient was significantly greater in NOD cells compared to *Idd9 *cells (p = 0.02). Colocalization appeared strongest in this peri-nuclear compartment, and therefore the redistribution of TRAF2 in *Idd9 *cells at 60 minutes after cytokine treatment is likely to account for the decreased M1 value. TNFR2 activation is associated with TRAF2 translocation to the ER [24]. The data therefore suggests that TNFR2 signaling is aberrantly sustained in NOD islet β cells following TNF exposure.

## Discussion

The results of this study suggest that resistance to autoimmune destruction can be controlled by the target islet cells, and the trait is genetically variable and determined by genes within the *Idd9 *interval. While Type 1 diabetes has traditionally been viewed as a disease of the immune system there is increasing support for the idea that islet responses are required for the progression to a destructive autoimmunity [25], and that defects in the target islet tissue may play a role in disease susceptibility. For example, in NOD mice blocking interferon signaling in β cells is protective against diabetes, particularly CD8-mediated diabetes, demonstrating the importance of islet cytokine-responsiveness for the induction of β cell death [6,26]. The ALR (alloxan-resistant) strain has a dominantly inherited systemic ability to dissipate free-radical stress. Islets from ALR mice are also resistant to diabetogenesis and CD8-mediated cytolysis, and appear able to retain insulin-secretory function following cytokine- or glucose-induced stress *in vitro *[27]. Transgenic expression of a B2M allele associated with diabetes protection has also been suggested to exert protection through non-hematopoetic cells [28]. Thus, islet mediated protection from autoimmunity has been demonstrated in several experimental models.

We found that splenocytes from *Idd9 *congenic mice possess the potential to transfer disease, demonstrating that the immune system in *Idd9 *congenic mice is fully capable of mounting a pathogenic anti-islet autoimmune response. Adoptive transfer to immunodeficient mice can reveal latent pathogenicity that may be less evident in immunosufficient hosts, and the onset of disease induction was delayed compared to NOD mice. Therefore, the possibility remains that an additional component of *Idd9 *genetic susceptibility affects the development of autoreactivity. At least three genes mediate diabetes protection in the *Idd9 *congenic strain used in this study [12], suggesting that other factors are likely to also be involved. Studies by other groups have found that CD8 T cell tolerance [29] and also T cell responsiveness to 4-1BB ligand [30] are affected by genes in the *Idd9 *interval. However, we have shown that *Idd9 *congenic mice depleted of immune cells retain their protection against disease in the presence of NOD-derived immune cells, and that transplanted islets from *Idd9 *congenic mice resist CD8-mediated destruction. The data therefore suggests that a component of diabetes resistance mediated by protective *Idd9 *genes maps to the target islets. It is conceivable that islet responses during the early stages of inflammation may influence the control of CD8 T cell tolerance described by Martinez et al [29]. Furthermore, the target-organ specificity of protection mediated by *Idd9 *genes is supported by a recent publication demonstrating that diabetes-protective B10 alleles at the *Idd9 *locus do not protect against autoimmunity in a non-islet target, in experimental autoimmune encephalitis [31].

It is interesting to consider how islet resistance to destruction by autoimmune CD8 T cells is related to our initial observations that the infiltrate in *Idd9 *congenic islets contains a reduced number of CD8 T cells. One potential explanation for the reduction in CD8 T cells is that their survival or retention in islets is dependent on cytokine-induced modifications that do not occur in the islets of *Idd9 *congenic mice. The actions of TNF and IFNγ induce a diverse program of changes in islets that can greatly increase their visibility to the immune system and support the accumulation of inflammatory cells, for example by the release of chemokines and cytokines. Protective cytokine responses in *Idd9 *islets may inhibit a range of pro-inflammatory changes that occur in NOD islets to promote the transition from islet infiltration to a destructive autoimmunity.

Both islet cell death and Fas expression are enhanced in NOD compared to *Idd9 *islets following exposure to the inflammatory cytokines TNF and IFNγ *in vitro*, suggesting that NOD β cells are hyper-responsive to these cytokines. NOD mice are known to express an allelic variant of one of the receptors for TNF, *TNFR2*, that is distinct from that expressed by the diabetes resistant C57BL6 strain [17]. *TNFR2 *maps within the *Idd9 *congenic interval and is therefore a plausible candidate gene for the *Idd9*-mediated control of TNF/IFNγ responsiveness and islet resistance [12]. We found that TNFR2 mediates the induction of Fas expression by islet β cells following cytokine exposure. Furthermore, TNFR2 inhibition was ineffective at reducing Fas expression in NOD β cells, whereas *Idd9 *islets were responsive to the antibody treatment. This suggests that TNFR2 signal termination may be impaired in NOD mice. TNFR2 signaling depends on recruitment of the TRAF2 adaptor protein. TNFR2 activation is followed by translocation of TRAF2 to the ER, where it is ubiqutinated and degraded resulting in signal termination [24,32]. We observed this process in *Idd9 *islets, which showed only low levels of colocalized TNFR2 and TRAF2 after 60 minutes of cytokine exposure. However, in NOD islet cells TRAF2 was found to remain associated with TNFR2. NOD β cells therefore appear to exhibit impaired termination of TNFR2 signaling that results in exaggerated cytokine responsiveness as evidenced by increased Fas receptor expression and cytokine induced death.

The cytoplasmic C436Y mutation in the NOD isoform of TNFR2 lies adjacent to the binding site for TRAF2 [22]. Structures sufficiently similar to the TNFR2 C436Y region to precisely model the effect of this mutation on TRAF2 binding are not currently available. However, examination of published structures for a TNFR2 peptide bound to TRAF2 (1CA9), and a LTβ R peptide bound to TRAF3 (1RF3) [33,34] suggests that residue 436 is located at the surface of the TNFR2 protein, and supports the hypothesis that introduction of tyrosine at this position may alter the stability of the binding interface between TRAF2 and TNFR2. One potential rationale for the aberrantly sustained signaling by the NOD TNFR2 isoform is that TRAF2 binding is more stabilized and induces stronger pro-inflammatory responses.

The molecular pathway to islet cell death mediated through TNFR2 is also a matter of interest. TNFR2 signaling can activate NF-kB through TRAF2. It has been shown in human islets that cytokine-induction of Fas expression is associated with NF-kB activation [35]. While TNF signaling through TNFR2 may affect the internal sensitivity of β cells to cell death, TNFR2 activation of NF-kB may act primarily to induce cellular changes that promote islet inflammation and susceptibility to CTL killing. TNFR2-mediated Fas induction on β cells, which is heightened in NOD mice, may promote islet sensitivity to CD8-mediated destruction. While TNFR1-dependent islet responsiveness has been shown to be critical for islet destruction by CD4 T cells [36], islet TNFR2 responses were not required for this CD4-mediated diabetes. Islet TNFR2 may therefore function primarily in licensing CD8 cytotoxicity. It has been proposed that avidity maturation of the low affinity self-reactive CD8 response may be dependent on early islet damage mediated by death receptors such as Fas [37], and potentially other receptors such as HVEM/TRAIL-R [38,39] could also be involved. However, Fas-deficient islets transplanted into wild type NOD mice are only slightly protected against destruction [40], and Fas deficient islets are also not protected in CD8-mediated TCR transgenic diabetes models [5,7]. Blocking multiple TNFR molecules increases diabetes protection, for example, by overexpression in islets of dominant negative FADD, the adaptor protein used by multiple death receptors to recruit and activate caspase-8 [41]. Furthermore, islet overexpression of decoy-receptor 3 (DCR3), that inhibits Fas, LTβ R and DR3 signaling, protects against diabetes and also reduces insulitis [42]. However, the program of cytokine-induced changes that affect islet infiltration and survival is likely to be wide-ranging, and the control of death receptor expression perhaps only one aspect of this. Further studies will be required to test the precise effects of the NOD TNFR2 mutations on TRAF2-dependent signaling and in mediating downstream effects on islet survival.

TNFR2 is also expressed on T cells following activation and is thought to act as a co-stimulatory molecule [43-46]. We have not addressed the effect of NOD TNFR2 mutations in T cells, but the signaling pathways activated by members of the TNFRSF appear to be cell and context dependent and it is possible that TNFR2 plays quite distinct roles in lymphoid and target cells. While TNFR2-deficient NOD mice are not protected against diabetes [47] the effect of TNFR2-deficiency in immune cells may mask the role of TNFR2-deficiency in islets. Indeed, it has been shown that systemic TNFR2-deficiency increases effector CD8 responses [48].

*TNFR2 *polymorphisms in human populations have been associated with susceptibility to inflammatory disease, in particular rheumatoid arthritis, systemic lupus erythematosus and Crohn's disease [21,49]. This supports there being an important role for TNFR2 in determining TNF responses and inflammatory disease pathogenesis, and further genetic and functional experiments will be required to test the importance of *TNFR2 *variants in controlling islet responses and diabetes susceptibility.

## Materials and methods

### Animals

NOD.B10*Idd9*R28 mice (line 1104) containing B10 genome across all three *Idd9.1/9.2/9.3 *intervals (48 cM), referred to here as *Idd9 *congenic mice, were obtained from Taconic Farms (*Idd9*R28, line 1104) and maintained in the rodent breeding colony at The Scripps Research Institute (TSRI). NOD and NOD*Scid *mice were obtained from the rodent breeding colony at TSRI. 8.3NOD*Scid *mice [13] were kindly provided by Dr. Pere Santamaria (University of Calgary), and a colony is maintained at TSRI. NOD.IL4KO mice were obtained from Jackson (stock 004222) and crossed with *Idd9 *congenic mice. NOD, NODIL4KO, *Idd9 *and *Idd9*IL4KO mice were generated by intercross of appropriate progeny and used as internal controls. Fluorescent genotyping using the polymorphic markers *D4Mit63*, *D4Mit72*, *D4Mit28 *and *D4Mit180 *was used to ensure retention of the entire *Idd9 *congenic interval during intercross. B6.TNFR2KO mice were obtained from Jackson (stock 002620). Female mice were used for all experiments. All live animal experiments were approved by the Institutional Animal Care and Use Committee (IACUC) and the Animal Research Committee (ARC) and were conducted in accordance with institutional guidelines for animal care and use.

### Diabetes Incidence

Diabetes incidence was determined by monitoring blood glucose levels weekly using Glucometer Elite strips. Mice with two successive blood glucose levels greater than 300 mg/dl were considered diabetic.

### Isolation of leukocytes from pancreatic islet tissue

Pancreas tissue was taken from 12 week old *Idd9 *congenic and NOD mice and, after removal of all panLN, immediately cut into small pieces and digested in 1 mg/ml collagenase P (Roche) in complete DMEM for 20 minutes at 37° with agitation. After washing, the digested pancreas was layered over Histopaque 1077 (Sigma) and the islets recovered from the interface after centrifugation. Following further washes, the islets were treated with 0.05 % trypsin/EDTA (Gibco) to create a single cell suspension. Leukocytes released from the trypsinized islets were incubated in DMEM at 37° for between 60 and 90 minutes to allow recycling of cleaved surface molecules. Cells were then counted by trypan blue exclusion and stained for analysis by flow cytometry. An antibody to CD45 was included in the staining in order to compare leukocyte subpopulations as a percentage of infiltrating cells, independently of the extent of infiltration. All antibodies used for flow cytometry were obtained from BD Pharmingen. Staining was performed according to standard procedures. A FACSCaliber dual laser cytometer was used in conjunction with CellQuest software for data acquisition and analysis.

### Immunohistochemistry

Freshly isolated pancreas tissue from 12–14 week old *Idd9 *congenic and NOD mice was snap frozen in OCT medium (CD8 staining) or fixed in 10 % NBF and paraffin-embedded (TNFR2 and TRAF2 staining). For CD8 staining, sections (10 μm) were cut from three different levels from each pancreas, each level separated by 300 μm, and air dried at room temperature overnight. Absolute ethanol was used for fixation, and 10 % normal goat serum, or BSA, was used as blocking agent. Anti-insulin (Dako, Carpinteria, 1/400) and anti-CD8 (BD Pharmingen, San Diego, 1/100) primary antibodies were incubated overnight at 4°. Texas Red conjugated anti-guinea pig (1/100) and Fluoroscein conjugated anti-rat (1/200) secondary antibodies (both from Vector) were incubated for 1 hour at room temperature in the dark. Topro-3 was used to stain nuclei and was added to anti-fade component A (Molecular Probes, Eugene OR) when mounting the slides. Slides were stored at -80° until analyzed using Bio-Rad MRC1024 laser scanning confocal microscope and 40 × oil objective lens, using Bio-Rad LaserSharp (v3.2) software to collect images. For colocalization studies, polyclonal goat anti-TNFR2 (R&D systems, 1/10 dilution), donkey anti-goat-fluoroscein (Molecular Probes, Eugene OR, 1/50), rabbit anti-TRAF2 (Leinco, 1/10-1/50 dilution), donkey anti-rabbit-Cy 5 (Jackson, 1/50), anti-insulin and donkey anti-guinea pig-Texas Red (Jackson, 1/50) were added sequentially for co-localization studies. DAPI was included in the mounting medium (Vector). Sections from B6.TNFR2KO mice stained with anti-TNFR2 antibody in parallel did not show specific staining. The TRAF2 antibody used for staining sections gave a single band when used for western blotting, demonstrating its specificity for the TRAF2 protein. Slides were stored at 4° until analyzed using a Bio-Rad (Zeiss) Radiance 2100 Rainbow laser scanning confocal microscope and 60× objective with 2.5× magnification. Zeiss LSM Examiner software used to determine M1 colocalization coefficient. Non-fluorescent TNFR2 and TRAF2 staining was performed on 12–14 week old pancreas sections as above except using DAB (Vector) to develop the signal.

### Adoptive transfer of 8.3NODScid splenocytes

Whole splenocytes from 4–6 week old 8.3NOD*Scid *mice were used in all experiments. Where CFSE labeling was required whole splenocytes were incubated in PBS at 5 × 10'7 cells/ml with 5 mM CFSE (Molecular Probes, Eugene OR) for 10 min at 37°. The cells were then washed twice in cold PBS and 20 million labeled cells were injected *iv *into recipient mice. The recipients of CFSE-labeled 8.3NOD*Scid *splenocytes were aged between 6 and 9 weeks of age, and were age-matched in each experiment. For the transplant experiments, Lympholyte M (Cedarlane Laboratories, Ontario, Canada) was used in some cases, according to the manufacturer's instructions, to remove dead cells and red blood cells, and 30 million unlabeled 8.3NOD*Scid *splenocytes were injected *iv *per mouse. A sample of the injected cells was labeled with antibodies to CD8 and Vβ 8.1/8.2 to confirm that all CD8 T cells injected were positive for the 8.3 TCR (CD8+ gated cells were 97–99 % Vβ 8.1/8.2+). To determine the activation phenotype the cells were also labeled with antibodies to CD44. The CD8+Vβ 8.1/8.2+ cells were typically approximately 40 % CD44hi. For CFSE transfer experiments panLN and ingLN from recipient mice were taken 4 days post-injection and single cell suspensions prepared in sterile filtered Hanks medium containing 5 % FBS. The cells were then stained with antibodies to CD8, CD44 and CD11a for analysis by flow cytometry.

### Adoptive transfer of diabetes

Donor splenocytes from 13 week old *Idd9 *congenic and NOD mice were lysed to remove red cells and injected *iv *into NOD*Scid *recipient mice. Either 20 million *Idd9 *congenic or NOD splenocytes were transferred into recipients, or 10 million of each population was co-transferred. Blood glucose levels were monitored for 13 weeks following transfer when all recipient mice were diabetic.

### Bone marrow chimeras

*Idd9 *congenic and NOD mice 6 weeks of age were lethally irradiated (950–1,000 Rad) and injected with bone marrow cells prepared from 5 week old NOD donors. Bone marrow was isolated from the leg bones of donor mice and resuspended at 50 million cells/ml. Following irradiation, recipients were injected iv with 10 million bone marrow cells in PBS, and maintained on antibiotics in the drinking water.

### Islet transplantation

Islets were hand picked from 5–7 week old *Idd9 *congenic and NOD donors. Typically 150–250 islets were isolated from each pancreas, and 9–12 donors of each strain were used for each experiment. Islets were isolated following pancreas inflation with collagenase through the common bile duct, as previously described [50]. Islets were then cultured in hydrophobic 35 mm Petri dishes for 7 days in 10 % CO2 at 37°, and the media changed every 3 days. RPMI 1640 containing 10 % FCS and supplemented with glutamine and antibiotics was used throughout the procedure. Approximately 250 islets were transplanted under the left kidney capsule of 11 week old NOD*Scid *recipients, as previously described [50]. The grafts were allowed to revascularize for seven days before transfer of splenocytes from 8.3NOD*Scid *mice. 5–7 days after splenocyte transfer the grafts were removed and fixed in 10 % NBF for paraffin embedding. Sections (4 μm) were cut and H&E stained for analysis of graft integrity and infiltration.

To score the extent of graft destruction adjacent sections were stained with anti-insulin and anti-glucagon (Dako, Carpinteria, 1/400) and scored as either healthy (normal distribution of predominantly insulin-positive cells with scattered glucagon-positive cells around the islet periphery) or collapsed/destroyed (few or no insulin-positive cells, and the predominance of glucagon-positive cells giving the islet a 'collapsed' appearance in glucagon-stained sections).

### Immunoblotting

Islets were isolated from 5 week old mice and allowed to recover in culture medium for 4–6 days before being stimulated with 1,000 U/ml IFNγ and 1,000 U/ml TNF (BD Pharmingen, San Diego, CA), with each cytokine individually, or with medium alone, for 2 days. Cells were lysed with RIPA buffer (containing 20 mmol/liter Tris, pH 7.5, 1 mmol/liter EDTA, 140 mmol/liter NaCl, 1% NP-40, 1 mmol/liter orthovanadate, 1 mmol/liter PMSF, and 10 μg/ml aprotinin) and 20–150 ug protein loaded per lane on a 12 % gel for western blot. Mouse anti-RIP (BD Transduction Labs, San Diego, CA) and rabbit anti-TRAF2 (Leinco, St. Louis, Missouri) antibodies were used for immunodetection (Cell Signaling Technology). All membranes were stripped and reblotted with a mouse mAb to actin to confirm equal protein loading (ICN Biomedicals). Densitometry was performed using ImageJ software (Nih).

### Islet cell death

To induce islet cell death, isolated islets from 5 week old donors were cultured with 1,000 U/ml INFγ and 1,000–5,000 U/ml TNF for 6 days. 50–100 islets were used per assay. Islets were then incubated with the Molecular Probes (Eugene, OR) Live/dead stain in RPMI-1640 for 45 minutes at 37'. Intact, unfixed islets were then transferred to microscope slides, excess buffer removed using a stretched Pasteur pipette, and a cover slip sealed over the islets. Confocal images using 40× oil immersion lens of Z-stack sections 50–100 μm through each islet were taken. The number of live and dead cells in each field was determined using ImageJ software.

### Islet cell flow cytometry

Hand-picked islets from 5 week old donor *Idd9 *congenic and NOD mice, or 4–6 month old B6 and B6.TNFR2KO mice, were cultured in complete RPMI-1640 media for 4–6 days to allow recovery and depletion of tissue-resident leukocytes. Islets were then treated with medium, IFNγ, TNF, or IFNγ +TNF for 48 hours. Cytokines were used at 1,000 U/ml unless otherwise stated. For blocking experiments islets were cultured with blocking anti-TNFR2 antibody (Pharmingen), or hamster IgG isotype control, for 1–2 hours prior to addition of cytokines. Islets were dispersed using 0.25% trypsin/EDTA for 4 min, washed and allowed to recover at 37' for 1 hour. Staining was carried out for 30 min at 4'C using PE-labeld anti-TNFR2 (clone HM102, Caltag), or with biotinylated anti-Fas (Jo2, Pharmingen) and SA-APC, or the relevant isotype control. 7AAD was added for 10 min at RT to exclude dead cells, and the samples analyzed immediately.

### Statistical tests

In all cases where a p value is shown, a two-tailed, unpaired Students' t-test was used to gauge significance, except for comparisons of diabetes incidences in which case the Kaplan-Meier survival test was used.

## Abbreviations

NOD, non-obese diabetic; panLN, pancreatic lymph node; ingLN, inguinal lymph node

## Competing interests

The author(s) declare that they have no competing interests.

## Authors' contributions

NH designed and carried out the experiments, with the technical assistance of AS and PS, and MS helped perform the transplant experiments. NS was involved in overseeing the project and in the design of experiments. EG performed the structural modelling of TNFR2/TRAF2 outlined in the discussion. The manuscript was written by NH and NS.

## Reviewers' comments

### Reviewers report 1

#### Dr Matthiasvon Herrath

La Jolla Institute for Allergy and immunology, San Diego, CA, United States

In this study, the author shows that genetic variation at the Idd9 diabetes susceptibility locus determines the resistance of pancreatic beta-cells to destruction. Susceptible NOD beta-cells show increased response to pro-inflammatory cytokines, enhanced cell death and increased Fas upregulation, which is mediated by TNFR2. TNFR2 lies within the Idd9 interval, and the diabetes-associated variant contains a mutation close to the TRAF2 binding site. In NOD beta-cells colocalization of TNFR2 with the adaptor TRAF2 is prolonged, thereby enhancing susceptibility of NOD beta-cells to destruction. The authors conclude that diabetes susceptibility is not only determined by the immune system but also by the susceptibility of the target tissue to destruction and suggests that protective islet TNFR2/TRAF2 signaling may result in resistance to islet destruction and diabetes.

#### General comments

This manuscript is interesting and very well written, the figures are clear (especially Figures 1C and 7D are very nice) and the conclusions correct. However, few specific points have to be addressed, which are listed below.

#### Specific points to be addressed

• Figure 1: The islet infiltrate studies have been performed with 12–14 week old NOD and Idd9 congenic mice, however, percentages of CD4 and CD8 T cells in peripheral blood and secondary lymphoid organs were derived from 6-week old mice. For better comparison, it would have been important to test secondary lymphoid organs and peripheral blood from 12–14 week old mice. Please add comment on whether the percentages of CD4 and CD8 T cells remained the same in these organs at 12–14 weeks of age.

##### Author's reply

We used young mice in these experiments to test whether there were differences in the % CD8 T cells in the steady state, before the onset of extensive insulitis, to try to distinguish against changes secondary to differences in insulitis. It is an important question though since the decrease in % CD8 T cells may occur systemically during the progression of autoimmunity rather than being specific to the islet environment. However, we have not found any evidence of this. We examined peripheral blood at 12 weeks of age to compare the % CD8 T cells in the circulation to that in the islet infiltrate, and observed a similar % CD8 T cells in the peripheral blood of NOD and *Idd9 *mice (NOD = 12.3 +/- 0.6, Idd9 = 13.6 +/- 0.9, n = 4 for each strain), and a similar % CD4 T cells (NOD = 46.4 +/- 1.1, *Idd9 *= 47.0 +/- 1.2, n = 4). There was also no difference in the % CD8 T cells in spleen at 9 or 20 weeks of age, although there was a slight increase in the % CD4 T cells in *Idd9 *mice. We have therefore added to the results section a sentence that no difference in the % CD8 T cell was observed systemically in older mice.

• Figure 2: It would have been better to perform the experiments described in Figure 2 with older recipients when insulitis is already strongly ongoing.

##### Author's reply

We again wanted to try to test for primary differences occurring in lymphoid organs before the effect of differences in insulitis influences events in these organs. It was also important to use younger mice because there is evidence that the pancreatic lymph nodes are not involved in diabetogenesis after 10 weeks of age since diabetes proceeds at the same rate if the panLN are removed at this time (Gagnerault et al, J Exp Med. 2002 Aug 5;196(3):369-77). In this respect, it is therefore appropriate to examine the effect of the *Idd9 *panLN environment on the priming of islet-specific CD8 T cell responses in recipients of an age where priming in the panLN is relevant.

• Figure 3A: The author states that there is no evidence that Idd9 congenic splenocytes are able to regulate diabetes induction (bottom of page 6). Which is the kinetic of diabetes incidence in NODScid recipients when only 10 million NOD splenocytes are transferred? If the recipients develop faster diabetes than in the presence of 10 million NOD splenocytes together with 10 million Idd9 splenocytes, then Idd9 splenocytes have a regulatory potential.

##### Author's reply

Transferring 10 million NOD splenocytes induces diabetes with slower kinetics than 10 million NOD + 10 million *Idd9 *splenocytes (67 % diabetes by 13 weeks post transfer compared to 100 % diabetes, respectively). 10 million *Idd9 *splenocytes induce diabetes in 50 % recipients at this timepoint (n = 6 each group). This further supports the conclusion that there is no evidence that *Idd9 *splenocytes have regulatory activity, and we have added a sentence to the results section describing this data.

• Figure 5A: The author explains in the text that TNFR2 mRNA expression has been shown to be induced in islet cells during diabetes progression (Ref. 18), so why do the authors then show TNFR2 flow cytometry stain in 5-week-old young mice instead of choosing older pre-diabetic mice as shown in Figure 5B with pancreas histology?

##### Author's reply

We used islets from young donors to test TNFR2 expression by flow cytometry partly in order to reduce the likelihood of contamination with TNFR2+ lymphocytes, but primarily since it is difficult to isolate islets when there is extensive insulitis present. This is true both in terms of isolating sufficient numbers of islets and also because they do not survive well in culture, presumably because of the large number of infiltrating cells. We treated the islets from young donors with cytokines in vitro to try to mimic the inflammation that would occur in vivo, but did not consistently detect an increase in the expression of TNFR2 following cytokine treatment.

• Figure 6: Please correct in the figure legend for Figure 6A that experiments were performed with B6 and not with Idd9 and NOD mice. Please use for Figures 6D and 6E and for the figure legend the same figure description (for example I+T+R2, I+T). Please explain why in Figure 6D and 6E IFN- treatment was taken as negative control and not medium only treatment as shown in Figures A-C.

##### Author's reply

We have corrected the labeling in the legend for Figure 6A, and also made the labeling in Figures 6D and 6E consistent. With regard to the use of medium or IFN alone as a control in these experiments, there is no difference in Fas expression between *Idd9 *and NOD in either medium treated or IFN-treated islets and IFN was used as a control in the later experiments only to reduce the number of variables that differ between control and experiment.

### Reviewers report 2

#### Harald Von Boehmer

Harvard Medical School, Dana-Farber Cancer Institute, Boston, MA, United States

• The manuscript by Hill et al. addresses the question whether resistance of target tissue (insulin-producing β cells) to autoimmune destruction contributes to diabetes susceptibility by analyzing islets in Idd9 congenic and NOD strains of mice. The authors show less abundance of CD8 T cells in Idd9 congenic islets, less induction of Fas by TNFRII signaling and note a difference in sequence in the TNFRII receptor in Idd9 congenic versus NOD mice adjacent to the TRAF2 binding site. They argue that this difference may be responsible for stronger TNFRII-TRAF2 association in NOD mice, resulting in stronger cytokine signaling which may be responsible (how?) for the increased percentage of CD8 T cells in NOD islet infiltrates. The authors leave open whether increased Fas expression in NOD islets directly contributes to increased cytotoxicity by CD8 T cells and it is still a matter of debate how much direct cytotoxicity by CD8 T cells contributes to the final stages of β cell destruction in the NOD model of type 1 diabetes.

##### Author's reply

We do not yet know how increased cytokine signaling in NOD islets may increase the % CD8 T cells. However, cytokine signalling in islets is known to increase expression of chemokines and cytokines that promote T cell recruitment and survival. It is also interesting to consider the possibility that since CD8 T cells seem to be specifically affected, it is perhaps direct interaction between target and CD8 T cells that results in increased T cell survival in NOD islets. We hope to address this question in future work.

We used Fas induction primarily as a marker of cytokine responsiveness. There is little evidence that Fas-deficiency alone protects islets against autoimmune destruction, but increased Fas expression is only one of a wide program of changes is induced in islets by cytokine exposure. The work we present here provides evidence that genetic variation in islet responses to cytokines contributes to the progression of insulitis to overt autoimmunity. The stage at which critical islet responses occur, and the downstream pathways that are key to diabetes protection, will be important questions to answer.

• The authors attempt to rule out that the relative resistance of Idd9 congenic mice has to do with genes expressed in hemopoietic cells but these studies are somewhat limited: hemopoietic cells from Idd9 congenic mice cause diabetes with a delayed onset in NOD mice and in experiments aimed at depleting host cells in Idd9 congenic mice injected with NOD hemopoietic cells 30% host cells were still present. This leaves open the question whether the Idd9 associated resistance becomes manifest in both β cells as well as hemopoietic cells. It is clear, however, that the TNFRII receptor gene is located in the Idd9 interval and hence represents at least one good candidate gene contributing to Idd9-mediated resistance. How the Idd9 TNFRII gene contributes to resistance is still largely an open question and may involve reduced chemokine, cytokine and/or Fas levels.

##### Author's reply

The *Idd9 *congenic interval in the strain used in these studies contains at least 3 distinct genes that influence diabetes susceptibility, and we agree that it is very possible that both islet and hematopoetic cells are affected by either the same or different genetic variants in the *Idd9 *interval.

• Minor point: Ref. 41 refers to a CD4 not CD8 T cell-mediated model of diabetes.

##### Author's reply

We have removed reference 41 from the discussion.

### Reviewers report 3

#### Ciriaco Piccirillo

**Dept. Microbiology and Immunology, McGill University, Montreal, Canada (nominated by Ethan Shevach, National Institute of Allergy and Infectious Diseases, National Institutes of Health Cellular Immunology Section, Laboratory of Immunology, Bethesda, MD United States)**.

This reviewer provided no comments for publication, due to personal circumstances.

## Supplementary Material

Additional File 1 Chimerism data for the experiment in Figure 3D. This figure shows the %Thy1.1+ cells within CD4 and CD8 T cell populations for secondary lymphoid organs in irradiated *Idd9 *(circles) and NOD (filled diamonds) recipients. No difference in %Thy1.1 (donor) cells between *Idd9 *congenic and NOD recipients is observed. Click here for file
