# Supplementary figures and images for: Resistance of the target islet tissue to autoimmune destruction contributes to genetic susceptibility in Type 1 diabetes
Source: Biol Direct. 2007 Jan 25;2:5. doi: 10.1186/1745-6150-2-5 (PMC1797159; doi:10.1186/1745-6150-2-5)

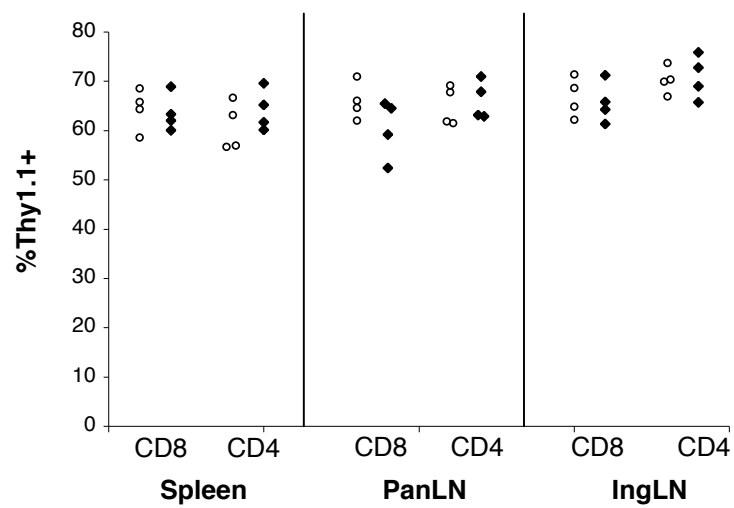

Supplement: Additional File 1 — Chimerism data for the experiment in Figure 3D. This figure shows the %Thy1.1+ cells within CD4 and CD8 T cell populations for secondary lymphoid organs in irradiated Idd9 (circles) and NOD (filled diamonds) recipients. No difference in %Thy1.1 (donor) cells between Idd9 congenic and NOD recipients is observed. [file 1745-6150-2-5-S1.pdf]
